# The effect of time constraints on resident performance in pediatric mock scenarios

**DOI:** 10.1186/s12909-025-07865-6

**Published:** 2025-11-11

**Authors:** Candace Collins, Madeline F. E. Parr, Tara Lozy, Amy Chirico

**Affiliations:** 1 Department of Pediatrics, Joseph M. Sanzari Children’s Hospital at Hackensack University Medical Center, Hackensack, NJ USA; 2 Center for Discovery and Innovation, Hackensack Meridian Health, Nutley, NJ USA; 3 Department of Pediatrics, Division of Critical Care, Joseph M. Sanzari Children’s Hospital at Hackensack University Medical Center, Hackensack, NJ USA; 4 Department of Pediatrics, Hackensack Meridian School of Medicine, Nutley, NJ USA

**Keywords:** Simulation, Mock code, Rapid response team, Time constraint

## Abstract

**Background:**

Hospital-based rapid response teams (RRTs) and code teams are expected to respond to acutely decompensating patients within a defined time. However, there is limited data regarding the effect of this defined response time on the performance of the activating team. Residents are commonly the first responders to acutely decompensating patients in the hospital setting and their ability to recognize the need for an RRT or code activation along with their skills in patient management until the responding team arrives appear to be important for patient safety. We sought to evaluate the effect of time constraints on the performance of pediatric residents during simulated clinical scenarios that require activation of the pediatric RRT or pediatric code team.

**Methods:**

We performed a single-center, prospective cohort study to analyze pediatric resident performance during low-fidelity simulated clinical scenarios involving acutely decompensating patients requiring pediatric RRT or pediatric code team activation. Simulated clinical scenarios were performed by residents without a time constraint (pre) and again with a time constraint (post) and residents were blinded to whether a time constraint had been applied. Statistical analysis was performed using a two-sided t-test to compare the number of interventions performed and time to activation of the pediatric RRT or code team for pre- vs. post-time constraint groups. Effect size was measured using Cohen’s d.

**Results:**

Implementing a time constraint did have a medium to large effect (Cohen’s d = 0.60) on pediatric resident performance and resulted in 6.2% increase in interventions performed in simulated clinical scenarios. Differences did exist based on the level of resident experience, with senior residents activating the pediatric RRT or pediatric code team faster than junior residents.

**Conclusion:**

Implementation of a time constraint did have a moderate to large effect on the performance of pediatric residents during simulated clinical scenarios involving hospitalized acutely decompensating patients.

**Supplementary Information:**

The online version contains supplementary material available at 10.1186/s12909-025-07865-6.

## Background

Rapid response teams, sometimes called medical emergency teams, were introduced in the 1990 s to improve patient outcomes for hospitalized critically ill patients by providing a dedicated team of trained healthcare providers [1]. These fast-responding, designated teams have since been incorporated nationally. The members of a rapid response team often include an intensive care unit physician, a nurse, a respiratory therapist, and, occasionally, a pharmacist [1, 2]. RRTs are typically activated when a patient requires additional medical support but has intact neurologic and cardiopulmonary function. Code teams differ from rapid response teams and are activated when a patient requires more intensive and immediate attention due to significant neurologic or cardiopulmonary compromise. The code team typically includes the same players in a rapid response team, but with the addition of nurses from surrounding units, an intensive care unit physician, and sometimes anesthesiologists [3].

Response times between RRT and code teams refers to the time that it takes for the RRT or code teams to respond after activating, or calling, the rapid response system. Individual institutions typically publish guidelines that include an expected response time for RRT and code teams to ensure patient safety. In our institution, an RRT team has 15 min to respond, and a code team has 5 min to respond. Previous data has shown an association between time to RRT activation and patient outcomes [4], making it vital for the primary medical team to activate these teams expeditiously. Time to initiation of compressions in CPR and timely patient care has significant impacts on patient outcomes. While not previously studied, it is logically necessary for the activating medical team to have the skills and knowledge required to stabilize these patients until the RRT or code team arrives. While simulation training appears to allow trainees to perform tasks more rapidly [5, 6], there is limited data regarding how the expected response times of RRT or code teams may impact the performance of the activating team.

Residents are commonly the first medical providers to respond to hospitalized acutely decompensating patients and are often responsible for activating a response system. At our institution, residents are most often the activators of the pediatric RRT and code teams. The expected response times of the RRT and code teams are 15 and five minutes, respectively, and response teams are trained with simulation scenarios. However, it is unclear how the arrival of an expected support team, such as a RRT or code team, affects the performance of the team who activates the rapid response system. Simulation learning has been highly successful in training residents to be proficient in procedural and hands-on skills, has a positive impact on time to complete tasks, and appears to improve patient outcomes [7, 8]. In a meta-analysis of simulation-based learning, Mazzone et al. found an approximately 15% faster time to complete procedures when trainees were taught using simulation rather than standard, non-hands-on learning [5]. While simulation training decreases time to perform tasks, there is limited insight into the impact of this time pressure in acute scenarios that would alter resident performance, either positively or negatively. Knowledge regarding this impact would guide the mitigation of lapses in patient care and provide the best possible patient outcomes. Simulated clinical scenarios are an integral aspect of residency training that prepares residents to manage these hospitalized acutely decompensating patients. This study aimed to evaluate the impact of time constraints on pediatric resident performance in simulated clinical scenarios in which pediatric residents managed acutely decompensating pediatric patients. The purpose of the time constraint is to provide a set end time to the scenario that would mimic the expected time frame that a resident would expect a RRT or code team to arrive to assist with a clinically deteriorating patient.

## Methods

A single-center, prospective cohort study was performed to analyze pediatric resident performance during low-fidelity simulated clinical scenarios involving acutely decompensating patients requiring pediatric RRT or pediatric code team activation. The study was reviewed and approved by the Institutional Review Board. Participating pediatric residents (subjects) were provided with an informed consent sheet for review prior to the session, and verbal consent was obtained prior to video recording via Zoom. Subjects were blinded with respect to the outcome measures of the study.

Fourteen scenarios were designed with varying degrees of difficulty targeted toward either junior-level (post-graduate year (PGY)−1 to early PGY-2) or senior-level (late PGY-2 to PGY-3) pediatric residents (Table 1). Scenario-specific scoring sheets were created prior to the sessions. They included points in patient evaluation (history and physical examination items), diagnostics, interventions, whether or not the pediatric RRT or pediatric code team was appropriately activated, and the time to activation of an RRT or code team. Activation time was defined as the time it took for a resident performing the mock scenario to verbalize the call to the RRT or code team. The independent variable was a time constraint. A time constraint was defined as the time after activation of RRT or code team to the time when the fictional RRT or code team would arrive. This time constraint was applied to the “post” group by stating the number of minutes the resident had to complete the scenario before starting. The primary outcome measure was the percentage of total possible points completed by subjects during the simulated scenarios. The secondary outcome measure was the time from the initiation of the scenario to the activation of the pediatric RRT or pediatric code team.

**Table 1:** Simulated scenarios performed pre- and post-time constraint

**Junior-level resident scenarios**	**Senior-level resident scenarios**
Anaphylaxis	Bronchiolitis with respiratory failure
Septic shock	Myocarditis
Opioid overdose	Multiorgan system failure
Acute gastrointestinal bleeding	Tricyclic antidepressant overdose
Acute hyperkalemia	Pericardial tamponade
Unstable supraventricular tachycardia	Electrolyte abnormalities following massive transfusion
Metabolic crisis	Mediastinal mass

Scenarios were designed and proctored by a single pediatric critical care attending physician to reduce variability in how scenarios were executed. Participating residents at the designated experience level volunteered to serve as scenario leaders. Leaders were instructed to ask for assistance from their observing co-residents (assistants) if needed. Assistants were instructed to join the scenario only when asked by the leader and only to provide information or assistance requested by the leader and not volunteer other information or assistance without prompting. All scenarios were video recorded via Zoom. Videos were reviewed and scored independently by the three investigators and averaged. Two points were awarded for each item completed by the leader. One point was awarded for each item completed by an assistant. The number of total possible points varied by scenario, so percentages of total possible points were designated for each scenario. Each scenario was performed first without disclosure of the duration of time for completion (pre-time constraint) during the academic year 2022–2023. The same scenarios were performed including disclosure of the duration of time for completion at the start of the scenario (post-time constraint) with new leaders with the same level of experience to the matched scenario during the academic year 2023–2024. Each video recording was reviewed again by the attending physician proctor just prior to the matched post-time constraint scenario to ensure uniformity. The duration of time for completion of the scenario was identical for each matched scenario pre- and post-time constraint and ranged from seven to 14 min.

Statistical analysis was performed using a two-sided t-test for comparison of the percentage of total possible points completed for each scenario pre- and post-time constraint. Secondary analysis was performed using a two-sided t-test to compare the time to activation of the pediatric RRT or code team for pre- vs. post-time constraint groups. Both analyses were performed with a significance threshold of 0.05. A one-way ANOVA with blocking was used to attain adjusted rates of completion and account for differences in the residents'experience levels when comparing the rate of completion or time to activation against time. Effect size was measured using Cohen's d.

## Results

**Fig. 1:**
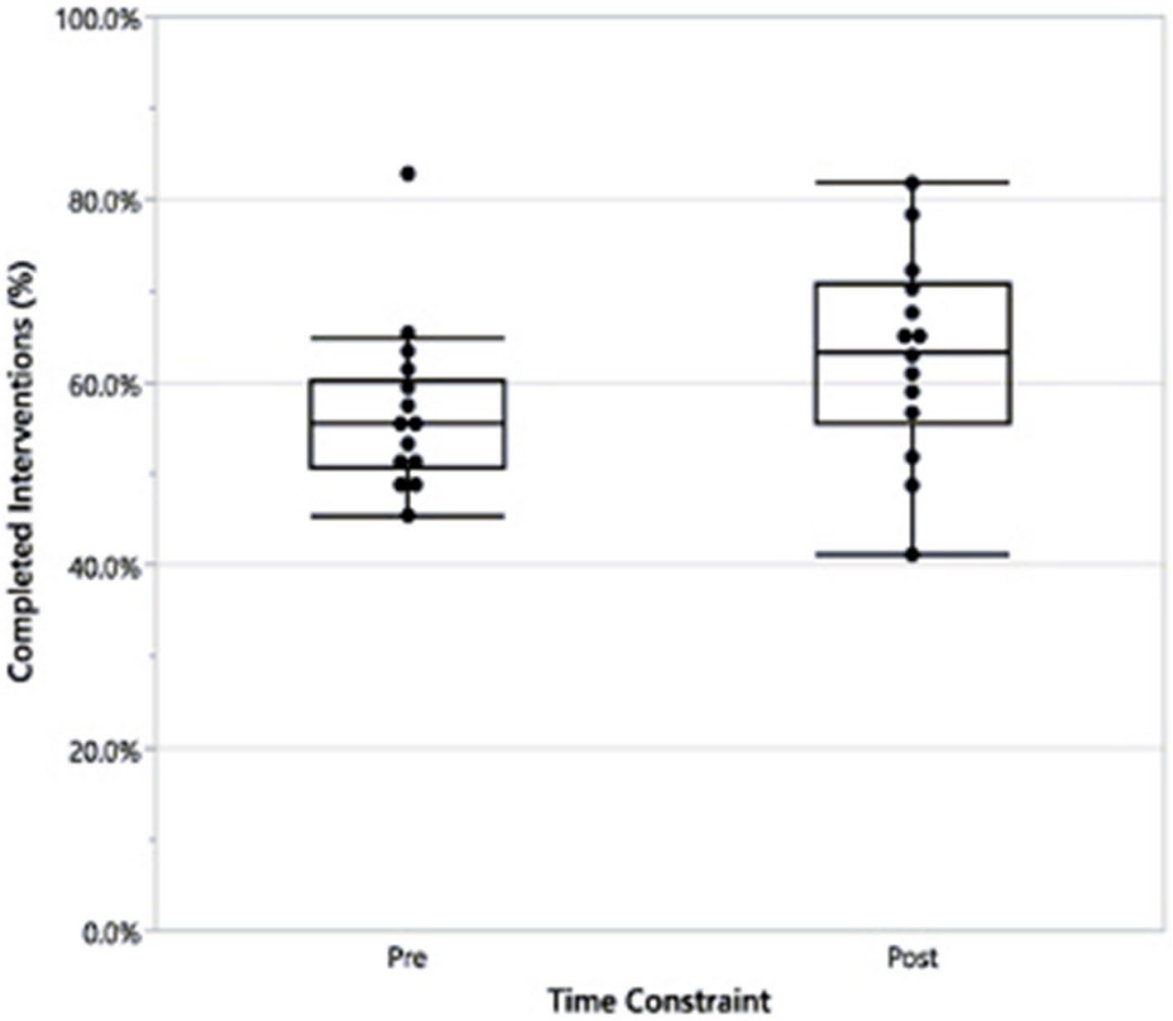
Percentage of total possible points performed without (pre) and with (post) an imposed time constraint

A total of 28 sessions were analyzed (14 pre-time constraint and 14 post-time constraint; Table 1). When comparing the percentage of total possible points completed, there was a 6.2% increase in raw points in the post-time constraint group. This difference reflected a medium to large effect size (Cohen’s d = 0.60); however, the difference was not statistically significant (*p* = 0.126, Fig. 1). Overall, junior-level pediatrics residents completed an average of 59.9% (± 13.5%) of total possible points in the pre-time constraint group and 70.9% (± 7.7%) of total possible points in the post-time constraint group (*p* = 0.113). Senior-level residents completed an average of 54.3% (± 3.9%) of total possible points in the pre-time constraint group and 56.8% (± 9.6%) of total possible points in the post-time constraint group (*p* = 0.516). While these differences were not statistically significant, the effect size was large for junior-level residents (Cohen’s d = 1.00) and small to medium for senior-level residents (Cohen’s d = 0.34).

**Fig. 2:**
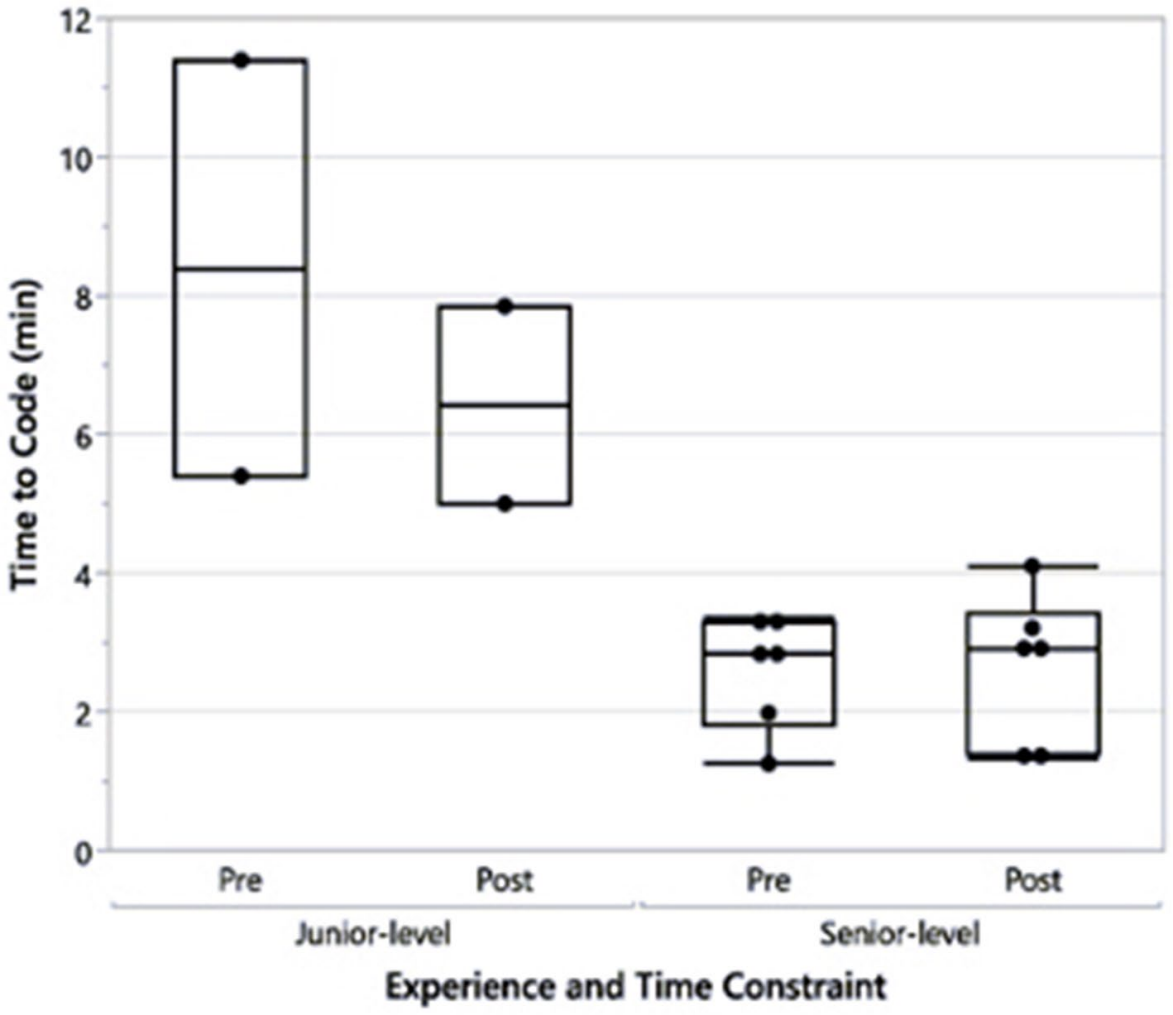
Time to the activation of the pediatric RRT or code team by resident experience level

There was no difference in time to activation of the pediatric RRT or pediatric code team between the pre- and post-time constraint groups. However, experience level did demonstrate a statistically significant difference with senior residents (late PGY-2 or PGY-3) activating the pediatric RRT or pediatric code team faster than junior residents regardless of the time constraint (*p* = < 0.001) (Fig. 2).

## Discussion

In this study, pediatric resident performance in simulated clinical scenarios was impacted by the implementation of a time constraint with a moderate to large effect (Cohen’s d = 0.60). The application of this time constraint resulted in a 6.2% increase in raw interventions performed, as measured with points scored in the time constraint group, but was found to be statistically insignificant. There are no previous studies that analyzed the effect of these time constraints on the activating team performance, but our data suggests that the time constraints imposed upon the responding team to ensure patient safety may not negatively impact patient care prior to their arrival. However, more research is required to evaluate the quality of the interventions performed.

Our results did demonstrate that senior-level residents activated the pediatric RRT or pediatric code team faster than junior residents. This likely reflects senior-level residents’ increased clinical experience, improving their abilities to recognize critically ill patients and escalate to higher levels of care. When studying resident management of simulated clinical experiences, Hunt et al. hypothesized that senior-level residents were more successful when they identified limitations and designated teams early, and their leadership of clinical scenarios impacted outcome [9]. However, our result may be confounded by the fact that senior-level resident scenarios were more complex and seemed more emergent or difficult, which prompted senior-level residents to call for help more quickly. While the results were not statistically significant, junior-level residents completed a higher percentage of total possible points in both pre- and post-time constraint compared to senior-level residents (59.9% pre and 70.9% post in junior-level vs. 54.3% pre and 56.8% post in senior-level). This may be attributable to the fact that the senior-level resident scenarios were more complex and therefore the difficulty level was higher and/or the total number of possible points was higher for senior-level resident scenarios based on this complexity and the number able to be completed was limited by the time constraint.

There are several limitations to this study. First, the power of the study is limited by the sample size with only 14 scenarios compared pre- and post-time constraint. Additionally, pediatric residents from a single center were evaluated. Continued evaluation including a larger group from more than one institution and other activating providers including bedside nurses, advanced practice nurses, and attending providers would improve generalizability. In addition, the study was not double-blinded. The residents who participated in the study were blinded to the outcome measures, but the outcome measures and time constraints were known to the investigators at the time of data collection. In addition, some of the junior residents in the pre-intervention period also performed sessions as senior residents in post- intervention period as the evaluations spanned over one year, but residents were assigned to lead different scenarios in each of the years. The scenarios in this study also utilized low-fidelity mannequin models, which may affect the realism of the scenario [10]. While the lack of realism may make it difficult to apply these results to real-life scenarios, high fidelity models may not be superior to low fidelity models in medical trainee knowledge acquisition [11]. In general, it can be challenging to extrapolate results from studies utilizing simulated clinical scenarios to real-life patient care scenarios. While preparing for acute situations prior to their occurrence is important, studying an activating team's performance in real-life patient care scenarios involving hospitalized acutely decompensating patients may provide the most accurate representation of a team's performance and highlight areas for improvement. It is possible that over time with increased experience with simulation scenarios, performance may trend toward improvement.

Further studies are necessary to enhance our understanding of the effect of time constraints imposed on the activating teams in both simulated and real-life scenarios. Understanding the psychological impact of time constraints on activating providers may also provide valuable insight into an individual's potentials and limitations. Investigating these impacts in future studies could lead to improved strategies for managing acute care scenarios and ultimately enhance patient care.

## Conclusion

Implementation of a time constraint had a moderate to large effect on the performance of pediatric residents during simulated clinical scenarios involving acutely decompensating hospitalized patients. Senior-level pediatric residents demonstrated a greater ability to recognize the need for activating a pediatric RRT or code team compared to junior-level residents. Additional research is required to more comprehensively understand the effect of response time on individuals activating the pediatric RRT or pediatric code team in both simulated and real-life scenarios.

## Supplementary Information

Supplementary Material 1.

## Data Availability

No datasets were generated or analysed during the current study.

## Abbreviation

RRT: Rapid response team
