# Supplementary material for: The effect of time constraints on resident performance in pediatric mock scenarios
Source: BMC Med Educ. 2025 Nov 11;25:1571. doi: 10.1186/s12909-025-07865-6 (PMC12606940; doi:10.1186/s12909-025-07865-6)
Supplement: Supplementary file 1 — Supplementary Material 1. [file 12909_2025_7865_MOESM1_ESM.pdf]

Supplementary Material: Sample Score Sheets for Pre-and Post- Intervention Scenarios

Example Junior Level Scenarios

GI Bleeding Score Sheet (Pre- and Post-Intervention)

**History**	**Performed (time)**	**Physical Exam**	**Performed (time)**	**Diagnostics**	**Performed (time)**	**Interventions**	**Performed (time)**
Weight		Vital signs		Place patient on a monitor		Ask about access	
PMH		General Appearance		Send labs		Request additional access	
Reason for admission		Cardiac		Send blood gas with lactate		Place patient on oxygen	
Prior course		Pulmonary		Send CBC		Specify route of oxygen delivery	
Ask about hematochezia/melena		Perfusion				Call senior resident for help	
		Repeat Vitals				Order IV fluid	
		Abdominal exam				Specify amount of IV fluid	
		Repeat physical exam after IV fluids				Specify type of IV fluid-isotonic crystalloid	
						Specify method of IV fluid administration-push-pull with 3-way stopcock	
						Ask for a second IV fluid bolus	
						Make a statement considering addition of vasoactive support	
						Order packed red blood cells	
						Consider activation of MHP/ABC cooler	
						Call blood bank to activate MHP/ABC cooler	
						Call pediatric RRT	
						Specify number for RRT	
						Summarize for incoming personnel	
						Call GI	

						Call Pediatric Surgery	
						Specify how to give pRBCs-push-pull with 3-way stopcock	
	5		8		4		20

Anaphylaxis Score Sheet (Pre- and Post-Intervention)

**History**	**Performed (time)**	**Physical Exam**	**Performed (time)**	**Diagnostics**	**Performed (time)**	**Interventions**	**Performed (time)**
Age and chief complaint		Vitals		Statement of concern about anaphylaxis		Removal of the inciting agent	
Reason for admission		General appearance		Statement regarding 2 system involvement in diagnosis of anaphylaxis		Place the patient on a monitor	
Past medical history		Cardiac exam		Ask about current access		Call for help (senior resident)	
Allergies		Lung exam		Place patient on monitor		Summarize case for incoming staff	
ED course		Perfusion				Place patient on oxygen	
Medications given		Skin exam				Specify type of oxygen delivery system	
Patient's weight		Mouth/lip exam				Request additional access	
		Repeat set of vitals				Order an IV fluid bolus	
						Specify type of fluid	
						Specify method of fluid administration	
						Order IM epinephrine	
						Specify epinephrine dose	
						Specify epinephrine concentration	
						Consider adjunctive therapy-diphenhydramine, steroids, H2 antagonists	

						Consider calling rapid response	
						Call RRT using 7911	
						Consider requirement of further respiratory support including intubation	
						Consider calling Anesthesia for intubation	
7		8		4		18	

<u>Example Senior Level Scenarios</u>

Myocarditis with Cardiac Arrest (Ventricular Tachycardia) Score Sheet (Pre- and Post-Intervention)

**History**	**Performed (time)**	**Physical Exam**	**Performed (time)**	**Diagnostics**	**Performed (time)**	**Interventions**	**Performed (time)**
Patient age		Vitals		Place patient on a monitor		Place patient on oxygen	
Patient weight		General appearance		Ask about access		Specify type of oxygen delivery device	
Reason for presentation to Emergency Department		Cardiac exam		Ask for labs		Request additional access	
Birth history		Lung exam		Include blood gas		Call pediatric code	
Past medical history		Perfusion		Include CMP (end organ function)		Call pediatric code correctly-7911	
ED course		Abdominal exam (liver)		Include troponin		Start chest compressions immediately	
Medications given		Repeat Vitals		Include BNP		Summarize case for incoming staff	
Ask about prior diagnostics		Repeat exam		Make a statement about possible etiology of arrest-myocarditis		Assign someone to bag mask ventilation	

Ask to see chest x-ray				Ask for input from others regarding etiology of arrest		Assign someone to chest compressions	
						Place patient on a backboard	
						Place defibrillator pads	
						Order code dose epinephrine	
						Correctly specify code dose epinephrine	
						Rhythm check	
						Correctly state rhythm	
						Order for defibrillation	
						Correctly order defibrillation dose (2J/kg)	
						Consider changing compressors	
						Consider antiarrhythmics might be needed	
9		8		9		19	

Tracheostomy Obstruction with Cardiac Arrest (Asystole) Score Sheet (Pre- and Post-Intervention)

**History**	**Performed (time)**	**Physical Exam**	**Performed (time)**	**Diagnostics**	**Performed (time)**	**Interventions**	**Performed (time)**
Patient age		Vitals		Place patient on a monitor		Apply oxygen	
Patient weight		General appearance		Ask about access		Specify mode of oxygen delivery	
Reason for admission		Cardiac exam		Ask for labs		Attempt to pass a suction catheter	
Birth history		Lung exam		Include blood gas		Attempt to bag ventilate trach	
Past medical history		Tracheostomy site exam		Include CMP (end organ function)		Request additional access	
ED course		Perfusion		Include lactate		Call pediatric code	

Medications given		Repeat vitals		Order chest xray		Call pediatric code correctly-7777	
Prior hospital course including chest x-ray		Repeat exam		Statement about possible etiology of arrest-tracheostomy obstruction		Start chest compressions immediately	
Home respiratory support				Ask for input from others regarding etiology of arrest		Summarize case for incoming staff	
Current respiratory support				Ask about end tidal CO2		Assign roles	
When tracheostomy placed						Place patient on a backboard	
Size and type of trach						Place defibrillator pads	
						Order code dose epinephrine	
						Correctly specify code dose epinephrine	
						Rhythm check	
						Correctly identify rhythm	
						Consider changing compressors	
						Remove trach	
						Occlude trach and bag mask ventilate OR place endotracheal tube in stoma and bag ventilate OR replace tracheostomy tube	
						Consider endotracheal intubation	
	12		8		10		20
